# Elektrocochleographie mit Trommelfellelektrode: Altersbezogene Referenzbereiche für die Schwindeldiagnostik

**DOI:** 10.1007/s00106-026-01725-5

**Published:** 2026-02-05

**Authors:** Tom Gawliczek, Miranda Claire Morrison, Katharine Keller, Athanasia Korda, Stefan Weder, Marco D. Caversaccio, Georgios Mantokoudis

**Affiliations:** Department of Otorhinolaryngology, Head and Neck Surgery, lnselspital, Bern University Hospital, University of Bern, Bern, Schweiz

**Keywords:** Referenzwerte, Altersgruppen, Elektrocochleographie, Morbus Menière, Reference values, Age groups, Electrocochleography, Morbus Menière

## Abstract

**Hintergrund:**

Die Elektrocochleographie (ECochG) erfasst u. a. elektrische Potenziale des Innenohrs und dient der Diagnostik von M. Menière. Für extratympanale Ableitungen fehlen bislang altersabhängige Referenzwerte, was die klinische Interpretation erschwert.

**Ziel der Arbeit:**

Ziel war die Erstellung altersstratifizierter Referenzbereiche für das Summationspotenzial(SP)/Aktionspotenzial(AP)-Amplituden- und -Flächenverhältnis zur nichtinvasiven Diagnostik bei Verdacht auf endolymphatischen Hydrops.

**Material und Methoden:**

Es wurden 47 Ohren von 26 normalhörenden Probanden im Alter von 20–70 Jahren mittels extratympanaler Trommelfellelektrode untersucht. SP- und AP-Potenziale wurden standardisiert abgeleitet, Amplituden- und Flächenverhältnis berechnet und alters- sowie seitenabhängig analysiert.

**Ergebnisse:**

Die Normbereiche lagen für das SP/AP-Amplitudenverhältnis bei 0,13–0,46 und für das SP/AP-Flächenverhältnis bei 0,72–1,72 (P5–P95). Es zeigten sich keine signifikanten Seitenunterschiede, jedoch ein leichter altersabhängiger Anstieg beider Parameter, ohne klinisch relevante Auswirkungen.

**Schlussfolgerung:**

Die extratympanale ECochG ermöglicht eine minimal invasive, zuverlässige Diagnostik mit altersstratifizierten Referenzwerten. Sie stellt eine praktikable Ergänzung zur Bildgebung bei M. Menière dar und erlaubt eine differenziertere Beurteilung pathologischer Innenohrfunktionen.

## Abstract (en)

**Background:**

Electrocochleography (ECochG) records electrical potentials of the inner ear and is used, among other applications, in the diagnostic work-up of Ménière’s disease. For extratympanic recordings, age-dependent reference values are currently lacking, which limits clinical interpretation.

**Objective:**

This study aims to establish age-stratified reference ranges for the summating potential (SP)/action potential (AP) amplitude and area ratios for noninvasive diagnostics in patients with suspected endolymphatic hydrops.

**Materials and methods:**

A total of 47 ears from 26 normal-hearing volunteers aged 20–70 years were examined using an extratympanic tympanic membrane electrode. The SP and AP potentials were recorded in a standardized manner. Amplitude and area ratios were calculated and analyzed with respect to age and laterality.

**Results:**

The reference ranges (P5–P95) were 0.13–0.46 for the SP/AP amplitude ratio and 0.72–1.72 for the SP/AP area ratio. No significant side differences were observed. A slight age-related increase in both parameters was detected, without a clinically relevant impact.

**Conclusion:**

Extratympanic ECochG enables a minimally invasive and reliable diagnostic approach with age-stratified reference values. It represents a practical adjunct to imaging in Ménière’s disease and allows a more differentiated assessment of pathological inner ear function.

Die Elektrocochleographie (ECochG) erfasst elektrische Potenziale des Innenohrs als Antwort auf akustische Reize und liefert objektive Informationen zur Cochlea- und Hörnervenfunktion. Klinisch wird die ECochG in einem breiten Anwendungsspektrum eingesetzt [1, 2]. Insbesondere wird die ECochG zur Diagnostik bei endolymphatischem Hydrops (M. Menière) eingesetzt und findet in jüngeren Anwendungsfeldern zunehmend Verwendung zur intra- und postoperativen Überprüfung der Implantat- und Systemintegration [3–6]. Ursprünglich wurde die ECochG über eine transtympanale Elektrode gemessen. Da dieses Verfahren invasiv ist, wurde es nicht flächendeckend in klinische Standards integriert; entsprechende Empfehlungen werden je nach Zentrum unterschiedlich umgesetzt [7]. Charakteristisch sind 2 Komponenten der ECochG, das Summationspotenzial (SP) und das Aktionspotenzial (AP); das SP-Signal entspricht der maximalen Deflexion der Basilarmembran, das AP entspricht funktionell der Welle I der auditorisch evozierten Hirnstammpotenziale (ABR) [3, 8]. Bezüglich der Ableitung ermöglicht eine extratympanale Trommelfellelektrode (Tympanic-Membrane[TM]-Elektrode) eine nichtinvasive, patientenfreundliche Messung [8]. In der klinischen Auswertung werden das SP/AP-Amplitudenverhältnis (erstmals beschrieben durch Coats 1981 [9]) und das SP/AP-Flächenverhältnis verwendet; beide können die Sensitivität der Diagnosestellung bei M. Menière erhöhen [12–14]. Transtympanale Elektroden liefern größere Amplituden, sind jedoch invasiv [10, 11].

Für die extratympanale TM-Ableitung liegen bislang nur begrenzte und teilweise heterogene Referenzdaten vor. Zusätzlich erschweren Unterschiede im Messaufbau und im Elektrodendesign den direkten Vergleich zwischen Studien. Frühere Arbeiten verfügten teils über hohe Stichprobengrößen, weisen jedoch methodische Variationen auf und liefern keine konsistent altersstratifizierten Normwerte. Damit bleibt unklar, wie sich SP- und AP-Parameter über die Lebensspanne verändern und welche altersabhängigen Referenzintervalle eine zuverlässige diagnostische Abgrenzung ermöglichen [15, 16].

Um diesen Ansatz weiterzuführen, verfolgt die vorliegende Arbeit das Ziel, erste altersbezogene Referenzintervalle für SP- und AP-Parameter abzuleiten und darauf aufbauend altersabhängige Grenzwerte vorzuschlagen. Damit sollen die bestehenden Lücken früherer Normwertstudien geschlossen und pathologische Muster – etwa bei endolymphatischem Hydrops – verlässlich von Normalbefunden abgegrenzt werden.

## Methodik

### Ethik

Die Studie wurde gemäß der Deklaration von Helsinki in der Fassung von 2013 sowie unter Beachtung der einschlägigen lokalen Regularien durchgeführt. Die Genehmigung der Kantonalen Ethikkommission Bern (KEK-Bern; #2022-01996) lag vor. Sämtliche Teilnehmenden wurden mündlich und schriftlich aufgeklärt und gaben vor Studieneinschluss ihr Einverständnis.

### Studiendesign und Teilnehmende

Es handelt sich um eine monozentrische, querschnittliche Normwertstudie, die am Schwindelzentrum des Inselspitals Bern (Universitätsspital Bern) durchgeführt wurde. Eingeschlossen wurden normalhörende Erwachsene mit Reintonschwellen von höchstens 25 dB HL im Frequenzbereich von 0,25 bis 6 kHz, intaktem Trommelfell und unauffälliger otologischer Anamnese; Personen mit akuter Otitis, mit Tympanostomie oder mit neurologischen Erkrankungen mit potenziellem Einfluss auf akustisch evozierte Potenziale wurden ausgeschlossen. An beiden Ohren wurde eine ECochG durchgeführt. Im Fall einer Kontraindikation erfolgte die Messung ausschließlich am gesunden Ohr. Die Rekrutierung erfolgte in Altersdekaden zwischen 20 und 70 Jahren.

Insgesamt nahmen 26 Personen an den Messungen teil (15 Frauen, 11 Männer). Das Durchschnittsalter betrug 38,7 Jahre (26–63 Jahre). Die größte Altersgruppe stellten die 30- bis 40-Jährigen mit 13 Personen (50 %). Weitere 5 Teilnehmende (19 %) waren 20–30 Jahre alt, 3 (12 %) 40–50 Jahre, 4 (15 %) 50–60 Jahre und eine Person (4 %) 60–70 Jahre. Die Geschlechterverteilung war mit 58 % Frauen sowie 42 % Männern ausgewogen. Es wurden insgesamt 47 Untersuchungen mittels ECochG durchgeführt – davon 23 an linken Ohren und 24 an rechten Ohren. Die Anzahl der gemessenen Ohren pro Altersgruppe ist in Abb. 4 dargestellt.

### Messaufbau und Messprotokoll

Die Messungen wurden in Rückenlage in einer ruhigen, abgedunkelten Umgebung mit geschlossenen Augen durchgeführt. Vor der Platzierung der TM-Elektrode (Sanibel™-TM-Elektrode, Fa. Interacoustics, Middelfart, Dänemark) und Applikation von leitfähigem Hydrogel erfolgten die Otoskopie und eine Ohrreinigung sowie eine lokale Oberflächenanästhesie des Trommelfells, um die Platzierung der Elektrode angenehm zu gestalten. Die Sanibel™-TM-Elektrode verfügt über ein nichtinvasives Design mit einer weichen, abgerundeten Spitze. Sie ist in eine Silikonhülle eingebettet und ermöglicht dadurch eine Platzierung am Trommelfell mit geringerer mechanischer Belastung im Vergleich zu invasiven Nadelelektroden. Die aktive TM-Elektrode wurde ipsilateral am Trommelfell positioniert, die Referenzelektrode sowie die Erdung wurden an die Stirn gesetzt; das Setup erfolgte gemäß den Empfehlungen des Herstellers (Gerät Eclipse, Fa. Interacoustics) für extratympanale ECochG-Messungen für beide Ohren.

Die Impedanzen der Hautelektroden wurden möglichst niedrig gehalten (Ziel ≤ 5 kOhm, interelektrodal im Idealfall ≤ 2–3 kOhm), und auch für die TM-Elektrode wurde eine geringe Impedanz zur Verbesserung des Signal-Rausch-Verhältnisses angestrebt. Als Stimulus wurden 100-µs-Klicks mit alternierender Polarität (Rarifikation, „rarefaction“/Kondensation, „condensation“) präsentiert. Die Stimulationsrate lag im üblichen Bereich von 7 bis 12 Reizen pro Sekunde (hier 11.3/s). Der Pegel wurde auf ein angenehmes Niveau zwischen 80 und 95 dB nHL eingestellt. Pro Mittelung wurden mindestens 2000 Einzelantworten aufgezeichnet. Das Zeitfenster reichte von −2 ms bis +5 ms. Als Standardschwelle für die Artefakterkennung wurde ±40 µV verwendet; Signale oberhalb dieser Schwelle wurden automatisch als Artefakte verworfen. Bei klinisch beobachteter, ausgeprägter Unruhe wurde die Artefaktschwelle in Ausnahmefällen auf maximal ±80 µV erhöht; gleichzeitig wurde die Anzahl der aufgenommenen Sweeps von 2000 auf 3000 gesteigert, um trotz erhöhter Artefaktlast eine stabile Mittelung und ausreichende Datenqualität zu gewährleisten. Rarefaktions- und Kondensations-Antworten wurden getrennt gemittelt und anschließend summiert, um die Unterdrückung des cochleären Mikrofonpotenzials zu verbessern und die Beurteilbarkeit von SP und AP zu erhöhen [1, 5]. Die Aufzeichnungen erfolgten mit einem klinischen Messsystem (Eclipse, Fa. Interacoustics); Filter- und Verstärkungseinstellungen wurden an das verwendete TM-Elektroden-Set angepasst und folgten den herstellerseitigen Empfehlungen (Hochpassfilter: 10-Hz-Tiefpassfilter; 5000 Hz).

### Definition und Berechnung der SP- und AP-Komponenten

Die Auswertung basierte auf 2 Kenngrößen: dem Verhältnis der Spitzenamplituden (Amplitudenverhältnis) und dem Verhältnis der integralen Flächen unter den Kurven (Flächenverhältnis) von SP und AP. Die Identifikation der Amplituden erfolgte durch 2 unabhängige Beurteiler; bei Divergenzen wurde eine dritte Fachperson zur Konsensfindung hinzugezogen. Das SP wurde als baselinenegative Verschiebung mit erstem negativem Spitzenmaximum im Zeitfenster von 0,5 bis 1,5 ms nach Stimulusbeginn definiert; das AP wurde im Zeitfenster von 1,2 bis 2,5 ms bestimmt [2, 12].

**Abb. 1:**
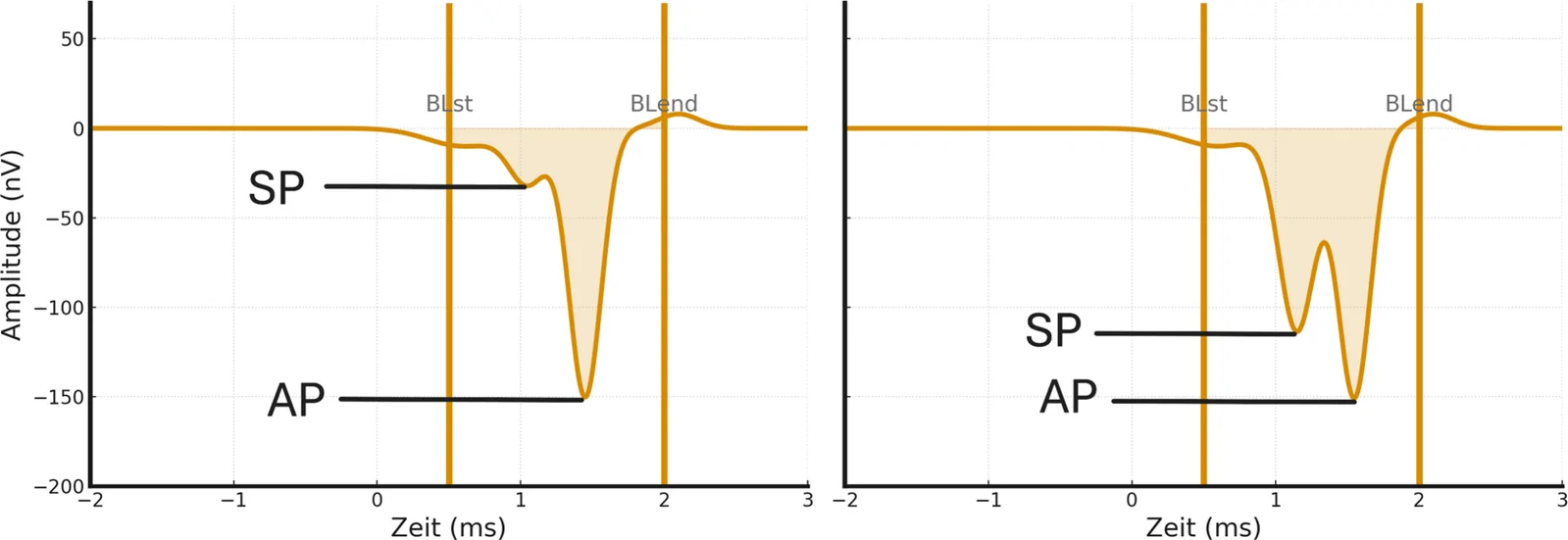
Beispiel einer Elektrocochleographie (ECochG) bei einer normalhörenden Person (*links*) und einer Person mit einem endolymphatischen Hydrops (*rechts*). Bildung des SP/AP-Verhältnisses durch Berechnung der Amplitude des SP relativ zur Amplitude des AP. Für die Flächenberechnung Ermittlung der Fläche unter der SP-Kurve im definierten Zeitfenster (*BLst *Baseline: Startpunkt, *BLend* Baseline: Endpunkt) und Betrachtung der Relation zur AP-Amplitude

Das Amplitudenverhältnis wurde gebildet, indem die Betragsspitzen von SP und AP ins Verhältnis gesetzt wurden. Das Flächenverhältnis wurde nach dem Vorgehen von Ferraro und Tibbils berechnet: Hierzu wurden für SP und AP a priori Verhältnisfenster festgelegt, die sich an den lokalen Extrema orientierten und gegenüber der Baseline (Mittelwert im Prä-Stimulus-Fenster) integriert wurden; die resultierenden Flächen für SP und AP wurden anschließend in Relation gesetzt [12–14]. In Abb. 1 ist das Beispiel der ECochG einer gesunden, normalhörenden Person (Abb. 1, links) dem Beispiel einer Person mit einem endolymphatischen Hydrops (Abb. 1, rechts) gegenübergestellt.

### Datenanalyse

Die Datenauswertung erfolgte verteilungsrobust. Das SP/AP-Flächenverhältnis unter der Kurve und das SP/AP-Amplitudenverhältnis wurden pro Ohr (links/rechts) mithilfe der Software EP (Fa. Interacoustics) berechnet. Hierzu wurden die Baseline sowie die Peaks des Summationspotenzials und des Aktionspotenzials markiert und daraus das Verhältnis für Amplitude und Fläche ermittelt. Seitenunterschiede wurden gepaart mit dem Wilcoxon-Vorzeichen-Rangtest (zweiseitig) geprüft. Alterszusammenhänge wurden mittels Spearman-Rangkorrelation und robuster Theil-Sen-Steigung quantifiziert; in den Scatterplots werden zusätzlich Bestimmtheitsmaß (R^2^) und Signifikanz angegeben (*p*-Wert). Der Methodenvergleich wird mithilfe von Boxplots dargestellt, die den Median, den Interquartilsabstand (IQR), das 5. und 95. Perzentil sowie die 95 %-Konfidenzintervalle enthalten. Das Signifikanzniveau war auf 0,05 festgelegt; bei Mehrfachtests wurde, falls nötig, nach Holm korrigiert.

## Ergebnisse

**Abb. 2:**
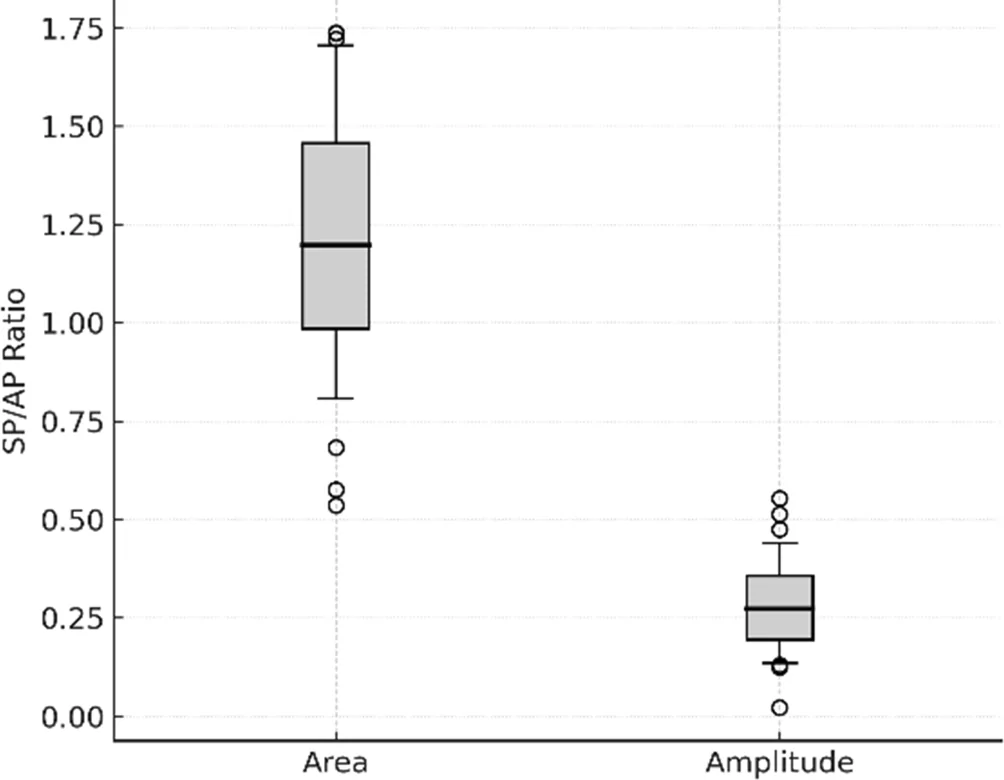
Altersunabhängige Darstellung des SP/AP-Verhältnisses (P5–P95) für die beiden Methoden *Area *(Fläche) und *Amplitude* mittels Boxplot. *Horizontale Linie *Median, *Kasten* Interquartilsintervall (IQR), *Whiskers* 1,5-faches IQR, *Kreise *Ausreißer

Zwischen linkem und rechtem Ohr zeigten sich keine signifikanten Unterschiede (Wilcoxon-Test) in Bezug auf die gemessenen SP/AP-Kenngrößen – weder für das SP/AP-Amplitudenverhältnis (*p* = 0,892) noch für das SP/AP-Flächenverhältnis (*p* = 0,785). In der altersunabhängigen Gegenüberstellung der beiden Verfahren (Abb. 2) zeigte das SP/AP-Amplitudenverhältnis eine engere Verteilung mit einem Median von 0,273 (IQR 0,19–0,36) und einem Normbereich (P5–P95) von 0,13–0,46. Das SP/AP-Flächenverhältnis zeigte eine größere Spannweite mit einem Median von 1,196 (IQR: 0,98–1,46) und einem Bereich für P5–P95 von 0,72–1,72.

**Abb. 3:**
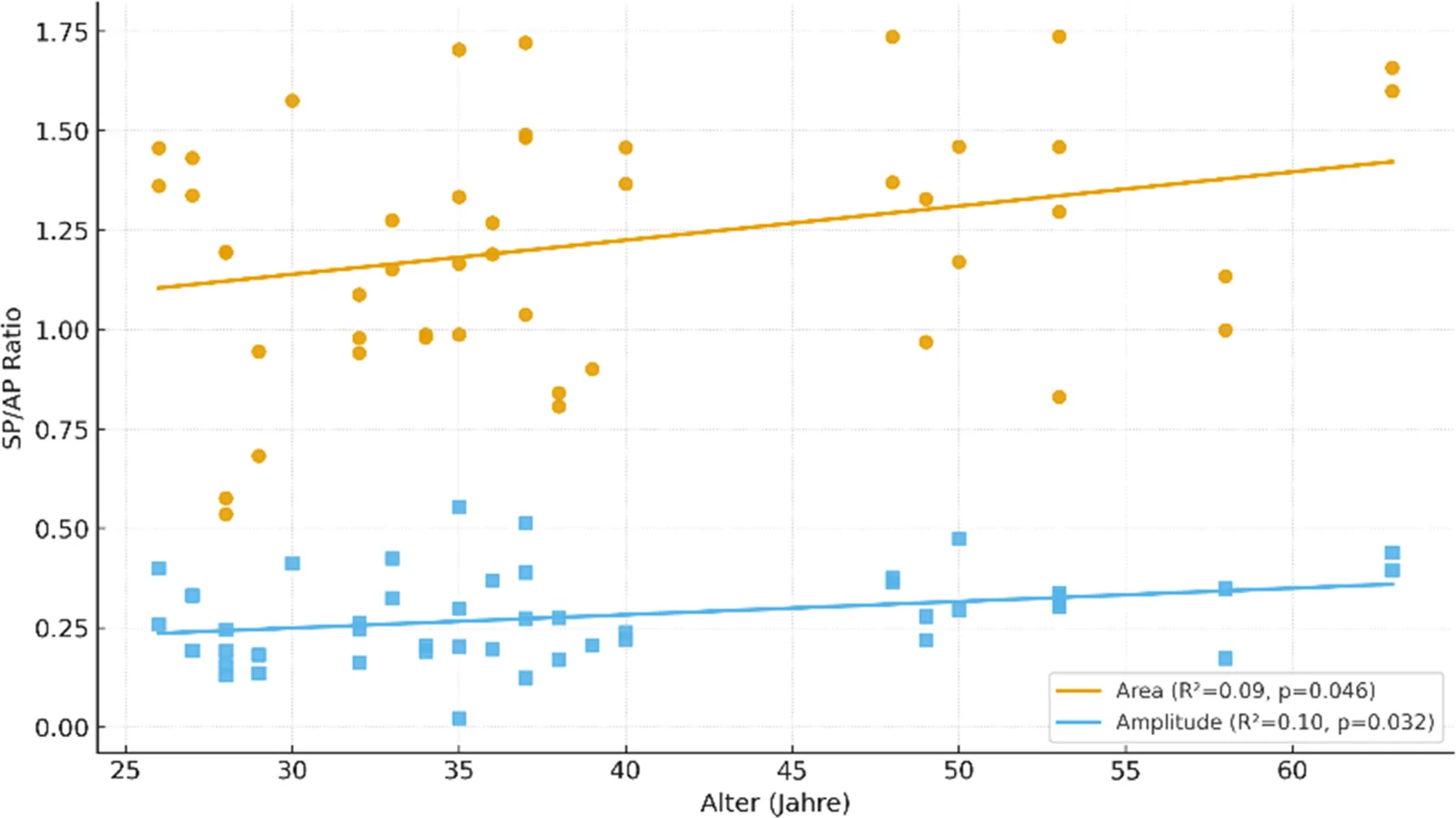
Altersabhängige Variation des SP/AP-Verhältnisses für die *Area*- und *Amplitude*-Methode (linkes sowie rechtes Ohr). Erläuterung s. Text. *x‑Achse *Alter in Jahren, *y‑Achse* SP/AP-Verhältnis, *Punkt *individueller Messwert; Regressionslinien mit den entsprechenden R^2^- und *p*-Werten: schwache, aber signifikante Zunahme des SP/AP-Verhältnisses mit steigendem Alter für beide Methoden. Altersabhängigkeit in der Berechnung der Amplitudenmethode

Die altersabhängige Betrachtung ergab für das Amplitudenverhältnis einen kleinen, aber robusten linearen Anstieg mit dem Alter (Abb. 3). Auf der x‑Achse ist das Alter in Jahren dargestellt, auf der y‑Achse das SP/AP-Verhältnis. Jeder Punkt repräsentiert einen individuellen Messwert; die Regressionslinien mit den entsprechenden R^2^- und *p*-Werten zeigen eine schwache, aber signifikante Zunahme des SP/AP-Verhältnisses mit steigendem Alter für beide Methoden. Die lineare Regression ergab eine Steigung von +0,0033 pro Jahr bei R^2^ = 0,10 und *p* = 0,032. Damit nimmt das Amplitudenverhältnis mit zunehmendem Alter leicht zu. Für das Flächenverhältnis zeigte sich ebenfalls eine tendenzielle Zunahme. Die Steigung betrug +0,0086/Jahr (R^2^ = 0,09; *p* = 0,046) und war damit richtungskonform, jedoch weniger deutlich ausgeprägt. Insgesamt spricht dies dafür, dass altersassoziierte Veränderungen ausgeprägter in der Amplituden- als in der Flächenberechnungsmethode sichtbar werden.

**Abb. 4:**
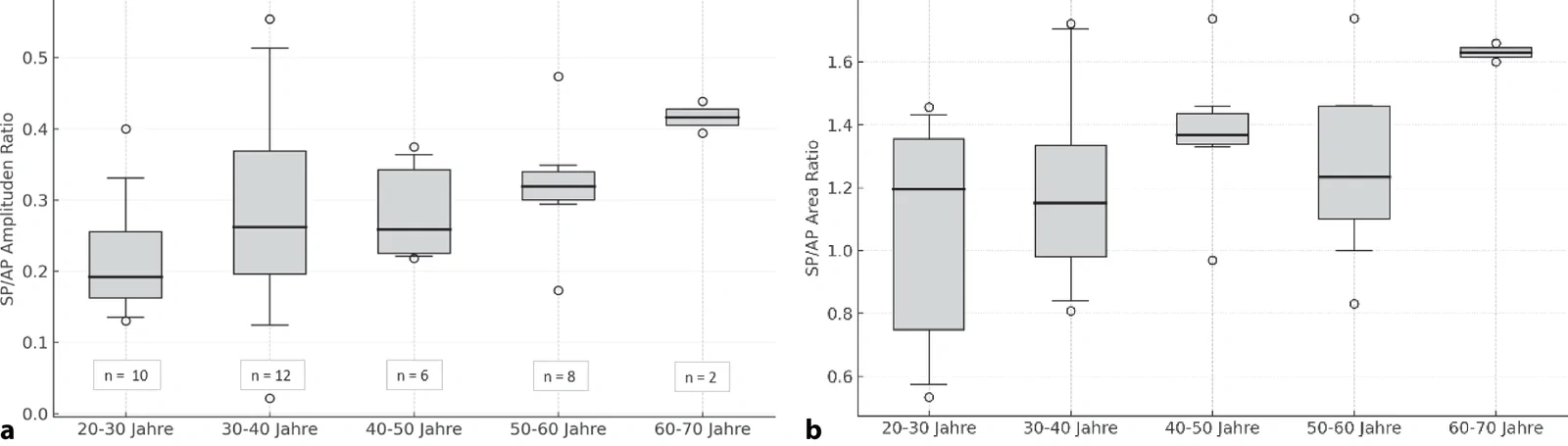
Altersabhängige Darstellung der Amplituden- (**a**) und Flächenberechnung (**b**) sowie der Anzahl (*n*) der pro Altersdekade erhobenen Elektrocochleographie(ECochG)-Messungen

Die Abb. 4 zeigt die dekadenspezifische Verteilung der Lage- und Streuungsmaße für jede Altersgruppe. Das Amplitudenverhältnis zeigt ansteigende Medianwerte von den 20–30 Jahren (0,192; P5–P95: 0,132–0,369) über 30–40 Jahre (0,262; 0,124–0,513) bis 50–60 Jahre (0,319; 0,215–0,430) und 60–70 Jahre (0,417; 0,396–0,437). Das Flächenverhältnis bleibt insgesamt breiter verteilt (z. B. 30–40 Jahre: Median 1,151; P5–P95: 0,841–1,704), mit erhöhten Medianen in den älteren Dekaden.

## Diskussion

In dieser Untersuchung wurde die ECochG mit einer extratympanalen Elektrode durchgeführt. Ziel war es, altersbezogene Normdaten zu erstellen, um die nichtinvasive Methode als ergänzende Diagnostik bei M. Menière – insbesondere bei Verdacht auf einen endolymphatischen Hydrops – zu unterstützen.

Die Ergebnisse lassen sich in 3 Kernaussagen zusammenfassen:

- Erstens zeigen die Parameter des Summationspotenzials (SP) und des Aktionspotenzials (AP) eine altersabhängige Korrelation, die als nicht klinisch relevant betrachtet werden kann.
- Zweitens finden sich keine seitenspezifischen Veränderungen der Normwerte in Abhängigkeit vom Alter.
- Drittens belegen die gewonnenen Normdaten die Eignung der extratympanalen ECochG als nichtinvasive, kostengünstige und machbare Zusatzuntersuchung im diagnostischen Vorgehen bei Verdacht auf M. Menière bzw. endolymphatischen Hydrops.

Ein wesentlicher Punkt für die klinische Interpretation betrifft die Abgrenzung zu pathologischen Befunden. Klassischerweise gelten SP/AP-Verhältnisse ab etwa 0,40 als auffällig und potenziell hinweisend auf einen endolymphatischen Hydrops. Das in dieser Studie beobachtete 95. Perzentil liegt jedoch mit 0,463 über diesem häufig verwendeten Grenzwert. Dadurch besteht das Risiko, dass bei alleiniger Anwendung eines fixen Grenzwerts von 0,40 vermehrt Normalbefunde als auffällig eingestuft würden. Dies unterstreicht die Bedeutung eigener, methodenspezifischer Referenzbereiche und die Vorsicht, frühere Grenzwerte nicht unkritisch zu übernehmen. Künftige Studien zur diagnostischen Genauigkeit – insbesondere solche mit expliziter Einbindung von Menière-Patienten und ROC-Analysen – werden erforderlich sein, um präzisere und klinisch valide Grenzwerte für die Differenzierung dieser Gruppen zu definieren.

Die Tab. 1 fasst normative Daten von ECochG-Messungen im Zusammenhang mit M. Menière zusammen. Dabei gibt es Unterschiede beim Messaufbau, der benutzten Elektrode und Stimulus. Die Studie von Grasel et al. (2017) zeigt vergleichbare normative Werte mit der vorliegenden Studie mit ähnlichem Versuchsaufbau [20].

**Tab. 1:** Normative Elektrocochleographie(ECochG)-Daten im Zusammenhang mit M. Menière

Autor (Jahr)	Anzahl *n* (Ohren)	Elektrodenart	Stimulation	Mittleres SP/AP	Oberer Grenzwert
Gibson et al. (1983) [17]	32	TT (promontoriale Nadelelektrode)	Klick 100 dB	**0,25**	**0,29**
Levine et al. (1992) [18]	26	ET (Trommelfellelektrode)	Klick 88 dB, ~10/s	**0,31**	**0,42** (+2 SD)
Roland et al. (1993) [19]	34	ET (Gehörgangselektrode, TIPtrode)	Klick 95 dB HL, ~10/s	**0,22**	**0,34**
Margolis et al. (1995) [15]	53	ET (Trommelfellelektrode)	Klick ~88 dB HL, ~10/s	**0,26**	**0,44** (+2 SD)
Grasel et al. (2017) [20]	129	ET (Trommelfellelektrode, TM)	Klick 90 dB HL, 11,3/s	**0,21**	**0,36** (95 %-Perz.)
Lamounier et al. (2017) [21]	24	ET (extratympanal)	Klick 99 dB HL, 11,3/s	**0,24**	**0,46** (+2 SD)
Maheu et al. (2017) [22]	18	ET (extratympanal)	Klick 90 dB HL, 7,1/s	**0,19**	**0,24** (+2 SD)
Gawliczek et al. (2025)	47	ET (extratympanale Nadelelektrode)	Klick 95 dB HL, 10,9/s	**0,273**	**0,463** (95 %-Perz.)

Beachtet werden sollte, dass unterschiedliche Studien teils verschiedene Stimulus- und Auswertungsparameter nutzen; extratympanale Ableitungen ergeben im Mittel etwas höhere SP/AP-Verhältnisse als transtympanale. Abnormal erhöhte SP/AP-Werte > ~0,40 gelten als Hinweis auf endolymphatischen Hydrops (M. Menière) [12, 15]

*95* *%-Perz. *95 %-Perzentil,* TT* transtympanal, *ET* extratympanal, *TM-Elektrode* tympanal (am Trommelfell), *TIPtrode* goldbeschichtete Ohrstöpselelektrode im Gehörgang, *SP/AP* Verhältnis von Summationspotenzial- zu Aktionspotenzialamplitude, *oberer Grenzwert* oberes Limit normaler SP/AP-Werte: Menière-Verdachtsgrenze, oft definiert als Mittelwert ± 2 Standardabweichungen (SD) oder 95. Perzentil des Kollektivs

Eine altersabhängige Analyse der SP/AP-Verhältnisse zeigte sowohl deskriptiv als auch in der linearen Regression einen kleinen, aber robusten Trend zu höheren Werten im höheren Lebensalter. Für das Amplitudenverhältnis ergab die lineare Regression einen leichten, jedoch statistisch signifikanten Anstieg mit zunehmendem Alter. Das Flächenverhältnis zeigte tendenziell erhöhten Mediane in den älteren Dekaden; die oberste Dekade ist allerdings durch kleine Fallzahl limitiert und daher vorsichtig zu interpretieren. Die begrenzte Stichprobengröße, insbesondere in den höheren Altersdekaden, erlaubt jedoch keine robuste Quantifizierung des Effekts und verhindert eine zuverlässige Ableitung eng definierter, dekadenspezifischer Normbereiche. Die beobachteten Effekte erklären lediglich etwa 10 % der Varianz, und die Perzentilbereiche der einzelnen Altersgruppen überlappen substanziell, sodass die aus der Gesamtstichprobe abgeleiteten Referenzwerte durch den Altersbias nur geringfügig beeinflusst werden dürften.

Die vorliegende Studie untersuchte ausschließlich gesunde und normalhörende Probanden. Daher ist der daraus abgeleitete Schwellenwert nicht als diagnostischer Cut-off zur Unterscheidung zwischen gesunden und an M. Menière erkrankten Personen zu interpretieren; entsprechende Grenzwerte können in einer betroffenen Population an anderer Stelle liegen. Zudem kann das ECochG-Signal bei Betroffenen mit M. Menière durch eine permanente Hörstörung, wie sie bei einer fortgeschrittenen Erkrankung auftreten kann, reduziert sein oder nicht messbar sein. Die hier gewonnenen Normdaten lassen sich nicht ohne Weiteres auf abweichende Stimuli, alternative Elektrodentypen oder unterschiedliche Messaufbauten übertragen; jede Konfiguration benötigt eigene Referenzwerte.

Bisher war der Einsatz von transtympanalen Elektroden beliebt, da die Signalamplituden aufgrund der Nähe der Elektrode zur Cochlea größer und robuster waren. Dieser Test hat sich allerdings nie flächendeckend durchgesetzt, da er invasiv ist und Betroffene trotz Anästhesie Schmerzen oder Unbehagen verspüren können. Eine Perforation des Trommelfells ist für die Patienten nachteilig, da mögliche Komplikationen nicht auszuschließen sind.

Bisher wurden extratympanale Messungen häufig als weniger robust eingeschätzt, da das abgegriffene Signal kleiner ist und mehr Mittelungen notwendig sind im Vergleich zu transtympanalen Messungen. Fortschritte im Elektrodendesign, wie etwa die in dieser Studie verwendete weiche Silikonspitze, ermöglichen einen minimal invasiven Kontakt zum Trommelfell und zugleich robuste Messungen. Außerdem ermöglichen Verbesserungen in der Signalverarbeitung und Filterung eine deutlich zuverlässigere Datenerhebung als in früheren Arbeiten. Trotz Einsatz einer Bildgebung mit Hydropssequenzen sind immer noch falsch-negative MRT-Befunde bei Betroffenen mit M. Menière möglich [23]. MRT-Aufnahmen mit Hydropssequenzen (Protokoll „locally enhanced inner ear“, LEIM) korrelieren besser mit der Hörschwelle bei fortgeschrittenen Stadien [24]. ECochG kann daher insbesondere in der frühen Diagnostik oder bei unklarem MRT-Befund einen wichtigen komplementären Beitrag leisten.

Die extratympanale ECochG ist ein nichtinvasives Verfahren zur ergänzenden Diagnostik bei M. Menière. Obwohl sich bei gesunden Personen altersabhängige Unterschiede in den gemessenen Signalen zeigen, sind diese klinisch nicht relevant. Weitere Studien mit größeren Kohorten und klar altersstratifizierten Auswertungen sind erforderlich, um den diagnostischen Stellenwert der extratympanalen ECochG bei M. Menière zuverlässiger zu bestimmen und zu prüfen, in welchem Ausmaß sich altersabhängige Veränderungen auf die Messergebnisse auswirken. Normative Daten variieren jedoch deutlich in Abhängigkeit vom Messaufbau – insbesondere vom Elektrodentyp (trans- vs. extratympanal), vom verwendeten Stimulus sowie von den Einstell- und Filterparametern des Messsystems. Deshalb ist es entscheidend, für jeden spezifischen Messaufbau eigene Referenzdaten zu erheben, bevor die ECochG bei Betroffenen mit einem M. Menière eingesetzt wird. Perspektivisch kann die ECochG als diagnostische Ergänzung zur Bildgebung beim endolymphatischen Hydrops dienen.

## Fazit für die Praxis

- Die Elektrocochleographie (ECochG) ist eine minimal invasive diagnostische Untersuchung.
- Für eine Trommelfellelektrode (TM) spezifische Referenzbereiche für das Summationspotenzial(SP)/Aktionspotenzial(AP)-Amplituden- und Flächenverhältnis ermöglichen eine schnelle Befundklassifikation.
- Altersstratifizierte Normtabellen und Nomogramme mit Normbändern erleichtern Entscheidungen.
- Für die Anwendung im klinischen Alltag ist wichtig zu beachten, dass jeder Messaufbau – abhängig von Elektrode, Gerät und Stimulusparametern – eigene Normwerte benötigt.

## Data Availability

Die erhobenen Datensätze können auf begründete Anfrage in anonymisierter Form beim korrespondierenden Autor angefordert werden.
